# Understanding and redressing imbalances for South–North collaborations in energy and development research

**DOI:** 10.14324/111.444/ucloe.1974

**Published:** 2025-10-23

**Authors:** Muez Ali, Tash Perros, Penlope Yaguma, Tiago B. Diniz, Lilia C. Couto, Harshavardhan Jatkar, Jennifer Cronin, Pamela Fennell, Alexandre Szklo, Yacob Mulugetta

**Affiliations:** 1 Bartlett School of Environment, Energy and Resources, University College London, UK; 2 Department of Civil, Environmental and Geomatic Engineering, University College London, UK; 3 Department of Science, Technology, Engineering and Public Policy, University College London, UK; 4 Eletrobras Eletronorte, Rio de Janeiro, Brazil; 5 Energy Planning Program, School of Engineering, COPPE, Universidade Federal do Rio de Janeiro, Brazil

**Keywords:** energy and development research, collaborations, inequality, Global South, Global North

## Abstract

The Global South–Global North divide is widely defined using the Brandt Line, which proposed a geographical divide between more developed countries in the North and less developed countries in the South. Inequities in South–North research collaborations manifest in different ways and at different stages. Many researchers engaged in energy and development research are involved in collaborative projects with research partners across the divide. To ensure success, these collaborations must be inclusive and balanced. Researchers and multilateral organisations are starting to take notice of the potential negative impacts of unbalanced research collaborations. Critical assessments of these imbalances are scarce and there is a knowledge gap of ways to create more inclusive environments that allow researchers from the Global South to contribute solutions for challenges in their local contexts. Through workshops and a survey of researchers engaged in energy and development research, this paper attempts to partially fill this gap by investigating the challenges in collaborative projects faced by researchers in the Global South and Global North. The main findings show significant differences in the research experience of the two groups of researchers with respect to administrative burdens, access to resources, research roles and communication. We present several recommendations for how to address the inequities in collaborative research projects.

## Introduction

Energy is a key concern for the global development agenda [1,2] but energy and development are intrinsically multi-faceted challenges [3,4]. Therefore, research that is equally multi-faceted, inclusive and interdisciplinary is required to deliver a just transition and affordable clean energy for billions of people. Many researchers and institutions involved in energy and development research are engaged in collaborative projects with colleagues from different parts of the world. The benefits of such research collaborations and co-operation are well understood [5,6], however, they also bring specific challenges. To realise their full potential, it is crucial that collaborations are designed to be inclusive and that responsibilities are balanced appropriately between the parties involved.

Balanced collaborations allow for equitable resource exchange between regions, help financially support underfunded regions and enhance the research process by ensuring research questions are contextually sensitive [7]. However, there is growing evidence of imbalances between Global North (GN) and Global South (GS) countries in energy and development research and knowledge production. Ali et al. [8] found that the number of publications, citations received and funding allocation for research seeking to influence energy policy in low-and-middle-income countries (LMICs) are disproportionately skewed towards high-income countries (HICs). The authors suggest that to be effective, collaborations should create room for researchers based in LMICs to lead knowledge creation and solution-finding for the challenges in their geographical, social and political context. Similarly, Cronin et al. [9] argue that a just 1.5 °C transition requires the pursuit of a research agenda that is interdisciplinary, embeds procedural justice where everyone’s views are heard and respected, and engages with different actor groups who may have contrasting perspectives and radical ideas, some of which may come from regions or disciplines that have previously been underrepresented in academic research.

The aim of this paper is to explore the challenges and imbalances in their collaborative projects and to co-create recommendations for improving collaborations in energy and development research. The paper uses findings from a short survey and a workshop conducted with researchers working on collaborative projects on developing countries. The paper uses anecdotal evidence from survey respondents and workshop participants and uses UK Research and Innovation (UKRI’s) research guidelines for collaborative projects as demonstrative examples. Due to resource limitations and limited representation in the workshops and survey responses, this paper does not present generalisable findings. Instead, it seeks to shed light on some of the nuances in collaborative research projects and present examples of some of the structural barriers to equal participation. For convenience, we use the terms ‘Global South’ and ‘Global North’ throughout the paper to indicate the positionality of researchers and institutions.

## The Global North–Global South divide

The GN–GS divide was first widely defined using the Brandt Line concept in the 1980s [10]. The Brandt Line proposed a geographical divide between more developed countries in the North and less developed countries in the South (see the Royal Geographical Society [11] for a visual representation of the Brandt Line). At the time, the general assertion was that wealthy countries were almost all located in the geographical north and poorer countries were almost all located in the geographical South (except Australia and New Zealand). However, the Brandt Line holds minimal significance today as some countries and regions within the GS (China, Singapore and others) have developed considerably.

Such geographical classifications have been constructed over historical, economic, political and ideological terms, with the phrase GS often employed to the same effect as the ‘third world’; a ‘metaphor for poverty, oppression, suffering and underdevelopment’ [12,13]. But increasing globalisation has resulted in strategic economic blocs, such as the BRICS (Brazil, Russia, India, China, South Africa), which has enabled global co-operation and economic integration of the world’s leading emerging economies [14]. This has blurred the line between the GN and GS, but it should be noted that there is still significant inequality between regions, based on social, health and economic indicators and dimensions of the human development index. In this sense, references to the GN and GS as described by the Brandt Line may still be loosely relevant, used in international relations and in defining the socio-economic realities in the GS, and for highlighting inequalities and political divisions between different regions [15].

Although not an accurate geographical representation of socio-economic development, the GN–GS distinction can still be useful. The gap between the world’s richest countries and poorest countries remains pervasive [16] and, despite efforts to end extreme poverty, and some progress in the South, the GN remains a dominant economic and political power [14]. In the context of these disparities, the concept of the GN–GS divide can be used in academic knowledge production to advocate for an equitable knowledge system that embraces knowledge forms and processes from scholars, actors and institutions that have been traditionally excluded [15,17]. Therefore, while we use the terms GN and GS in this study, we acknowledge their limitations and use the terms as proxies to refer to the power and wealth divisions between research partners loosely based in these regions.

### Imbalances in South–North energy and development research collaborations

In a global effort to achieve universal access to affordable, reliable, sustainable and modern energy by 2030 as described in Sustainable Development Goal 7 (SDG7), it is not only necessary to mobilise financial resources for developing countries, but also to ‘enhance North–South, South–South and triangular regional and international co-operation on and access to science, technology and innovation and enhance knowledge sharing on mutually agreed terms’ (UN General Assembly [1], p. 26). There have also been growing calls for capacity building and mobilisation in Africa and the GS to develop and enhance the skills needed to tackle the climate change emergency and promote Southern-led development agendas [18], and meaningful local community engagement to democratise energy solutions implemented in the GS [19].

Inequities in South–North research collaborations manifest in different ways and at different stages across the research process – be it at project ideation, project execution, funding and resourcing or publications. For example, the burdens and inconveniences of facilitating fieldwork activities are often placed on researchers from the GS. Tilley and Kalina [20] illustrate instances of the gatekeeping burden on African researchers, such as the expectation to broker entry, access and establishment of relationships with local communities, or arrange and facilitate travel and comfort for researchers from the GN, sometimes at the expense of their time and convenience. Others observe that some collaboration models and methodologies, such as ‘Collaboratory-action parachuting’ [19], a novel method that combines the strengths of parachute research (or helicopter research)^1^ and community engagement participatory approaches to respond to specific energy needs in humanitarian emergencies, can empower local communities and GS collaborators. Yet, other models perpetrate imperialist views and minimise the involvement of GS researchers and communities, much to the detriment of the goals of these collaborations.

South–North collaborations have historically featured power imbalances, where research partners in the GN play the role of providing funding and resources for collaborations, and leading research projects [18]. Energy and development research relating to LMICs is most often funded by institutions in the GN, and the resulting publications first-authored by researchers based in HICs, which could lead to biased perspectives [8]. GN researchers have been challenged to consider these power differences between ‘the researcher’ and ‘the researched’ and rethink their role in collaborative research projects implemented in the Global South, by asking: ‘Who benefits?’ throughout a project lifecycle. With the exception of China, funding tends to be heavily concentrated in GN countries, therefore publications are disproportionately authored by researchers from or based in those same countries. As a result, the solutions and recommendations that emerge from this research, whether technical, political or social, are likely to reflect the views of the GN, which often disregard valuable local knowledge and promote inappropriate solutions for low-income countries. For example, key differences of perspective often arise from issues such as allocation of the remaining carbon budget across regions, immediate development needs and emissions pathways [21].

There are many collaborative projects between research institutions in the GS and GN in the energy and development field. Many involve a space for reflection on their strengths and weaknesses, but there is a missing dimension: an open conversation about pervading imbalances and how collaborations can be improved in the context of uneven access to resources and differing research priorities. Addressing these issues would ensure that solutions to energy challenges and a low-carbon energy transition for the GS are tailored to local needs and opportunities, adapted to local geographies and make efficient use of the resources available in both the GS and GN.

There is a need to deliberately create room for researchers from the GS to contribute to knowledge and solution-finding for the challenges in their local geographies and to examine how imbalances in collaborations manifest and how best equity can be achieved. On top of that, a broader dialogue is needed, to understand where and how imbalances in collaborations occur and what role different stakeholders can play in alleviating them [20].

This research attempts to address this gap by understanding the challenges and imbalances in collaborative projects between the GS and GN in order to co-create recommendations for improving collaborations in energy and development research. This was achieved via three main actions: (1) creation of an inclusive forum for open discussion between researchers examining experiences of collaborations in energy and development research; (2) co-creation of recommendations for improving collaborations targeted at researchers and funders; and (3) establishment of a network of researchers to co-develop a strategy for improved South–North collaborations.

The article is structured as follows: the Methodology section presents the study’s methodology, and the Findings section presents and discusses the results. The Recommendations section provides recommendations targeted at different concerned stakeholder groups and suggests a direction for future research. The final section concludes.

## Methodology

This project used a mixed-methods approach to examine relevant stakeholders’ perceptions of working on South–North collaboration projects in energy and development. First, a two-stage methodology was adopted, starting with an online survey, which allowed the identification of aspects of collaborations worthy of further exploration. Second, two online participatory workshops were organised, which qualitatively delved into these aspects in more detail. Experts and researchers reflected on successful collaborations, the underlying reasons behind imbalances in collaborative projects, and other challenges faced by researchers in the GS. The dialogue examined collaborative research experiences, including when they worked best and how they could be improved, recommendations for improving collaborations, and overcoming barriers. The project built on the work of initiatives such as the Low Carbon Energy for Development Network [22], shedding light on the voices and views of researchers from the GS. Data collection took place from March to May 2022.

Data was collected via Google Forms and was hosted on a specially created website. The questions in the survey are provided in the Appendix. We explicitly targeted academic researchers working on energy and development with first-hand experience of South–North collaborations. Participants were recruited through social media (LinkedIn, Twitter, Facebook), topic-relevant email lists and personal networks.

The survey (n = 49) primarily asked questions about participants’ meta information (e.g., country of residence, country of research focus, funding sources), their experiences of South and North partner involvements in different stages of the project and of positive and negative aspects of South–North collaboration projects. Two open-ended questions explored the advantages and disadvantages of collaborative projects. The data collected was mostly quantitative and was analysed in Excel. At the end of the survey, respondents were asked if they wished to participate in a workshop to explore the topic further.

Those survey respondents who expressed further interest were invited to take part in a workshop. Additional participants were also recruited directly through personal networks to give a total of n = 17 participants (Table 1). Descriptive analysis of the survey data looked to identify challenges faced by researchers either side of the divide, the role of researchers in the different stages of a research project and the difference in responses between GS and GN researchers. The findings from the descriptive analysis informed the discussions in the workshops. The workshops were recorded, transcribed and analysed thematically. The two workshops were purposefully scheduled to accommodate a range of time zones. They took place on Zoom under the Chatham House Rule and lasted for an hour and a half each. The workshops began with a short presentation about the results of the survey. Breakout rooms were subsequently used to co-create recommendations on how to improve South–North projects.

**Table 1.** The countries of origin of the workshop participants (country of origin refers to country of residence)

Country of origin	Number of participants
GS	
Brazil	4
Ethiopia	2
India	2
Kenya	1
Myanmar	1
Sudan	1
Uganda	2
Zambia	1
Global North	
France	1
UK	2

In the group discussions, discussants were invited to express their views on structural barriers – defined as a policy or practice inherent to the research process that is a hindrance to a balanced collaboration experience and beyond the control of individual researchers – to equitable collaborations and the challenges faced by researchers in both the GS and the GN. Descriptive analysis of the survey data looked to identify challenges faced by researchers either side of the divide, the role of researchers in the different stages of a research project and the difference in responses between GS and GN researchers. The findings from the descriptive analysis informed the discussions in the workshop. The workshops were recorded, transcribed and analysed thematically.

One limitation of the study is that the workshops had significantly more researchers from the GS (Table 1). This meant that GS voices were overrepresented. Having said that, the insights from the workshops are still useful for the broader aim of the study, and the perspectives of GS researchers are still valid. The South–North distribution of participants could perhaps also indicate that GS researchers are more keen to engage in discussions on the topic.

## Findings

**Figure 1:**
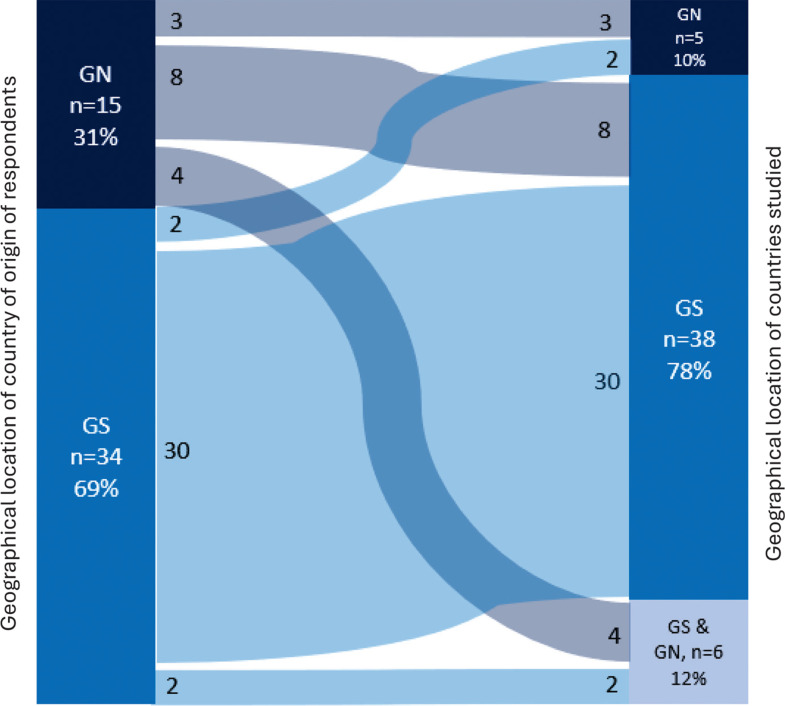
The countries studied by survey respondents.

The survey results show that most of the researchers from the GS primarily studied LMICs and only a small number researched HICs or both HICs and LMICs (Fig. 1). In contrast, half of the respondents from the GN only studied LMICs, a quarter studied both HICs and LMICs and a fifth specialise in HICs. Almost half of the survey respondents’ work was funded exclusively by institutions in the GN (49% of the respondents in the sample), 37% received funding only from the GS, 10% received funding from GS and GN funding bodies and 4% did not specify the source of funding. This reflects the larger proportion of GS participants.

Projects were funded by various types of institutions. The most common sources were research councils (27%), government departments or ministries (18%) and international organisations (14%). International and regional research institutions also featured as sources of funding. On the contrary, universities rarely funded energy and development research in the sample.

### Constraints from funders

One structural barrier identified by the discussants in the workshop is the way funding agencies operate. For example, a participant described how UKRI research grants require the principal investigator be based in an approved university or research organisation in the UK. The same grants allow for international co-investigators based in universities overseas, including developing countries, but under certain conditions. International research organisations, where necessary, are subject to eligibility checks on their capacity to conduct research, and the co-investigators identified for the project might only be eligible for salary costs if they are on a contract that expects them to ‘supplement their earning with external contracts’ (UKRI [23], p. 7) for a specific number of months in a year. A researcher on a contract that requires them to teach and conduct research throughout the year would not be eligible for salary costs. Such stipulations incentivise researchers to seek out more flexible contracts that could potentially hurt their career progression. And because grants are never guaranteed, this introduces income uncertainties for researchers in GS universities.

One researcher mentioned that funding bodies currently have no requirements for involving local researchers in the research outcomes of projects, such as including them as co-authors in publications. Having said that, the workshop discussants conceded that, for practical purposes, researchers should focus on barriers that are within their control instead of trying to tackle structural barriers, such as funding agency guidelines. For example, the design of a funding call can address some of the imbalances in research collaborations. One participant mentioned that senior academics and experts are sometimes invited to help design funding programmes, and that this is an opportunity to influence the type and structure of projects that receive funding.

**Figure 2:**
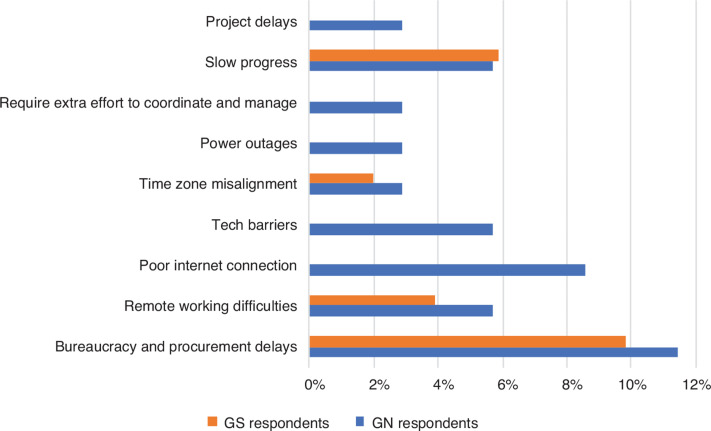
Administrative challenges faced by survey respondents (% of respondents).

Survey respondents agreed that collaborative projects suffer from bureaucracy, delays and remote working difficulties. And, as a result, progress is often slow. This was highlighted by a respondent from the GS, who cited frustrations about how bureaucratic red tape in funding processes leads to a lot of delays. Figure 2 shows that administrative difficulties seem more burdensome for GN respondents. One GN respondent explained that some of the factors that impact research performance that must be addressed include unstable internet connections, limited computational resources and power outages.

Based on the authors’ experiences, this is likely because these same technical issues – poor internet connection and power outages – are business-as-usual for respondents from the GS. In many developing countries, despite high internet penetration and increasing rates of electricity access, service disruptions due to political and economic factors are common in both urban and rural areas. These administrative barriers lead to inefficiencies in project implementation that threaten to detract from the positive aspects of collaborations. This is especially important for researchers in the GS who see various advantages to research collaborations, such as providing opportunities for access to funding and international recognition (see discussion in Research roles and power imbalances).

Another limitation affecting those engaged in international research collaborations is the complex and lengthy processes imposed by research institutions. The competitive academic environment in GN institutions pressures researchers to engage in research, policy impact and advocacy work all at once. The compulsion to publish in order to achieve certain milestones can shift the focus of researchers away from the content of the research towards career advancement and future opportunities for funding. When under pressure to publish, researchers might overlook issues of equity within the research design to achieve personal career goals [24]. So, how can we create balanced, equitable co-ownership over projects between GS and GN collaborators? A notable attempt mentioned by one of the participants in the workshop is the UK’s Global Challenges Research Fund (GCRF) established in 2015 to fund research on the most significant and complex problems faced by the developing world’ (UKRI [25], p. 3). GCRF provides specific funding criteria for partnering up with researchers from developing countries. On the nature of research, GCRF stipulates that research should ‘reflect local priorities’, and when conducting the research, ‘Southern partners [must] play a leading role in problem identification’ ([26], p. 516). The funding criteria also require ‘two-way research capacity development’ and that partnerships are long term, not project specific [26].

### Access to resources

**Figure 3:**
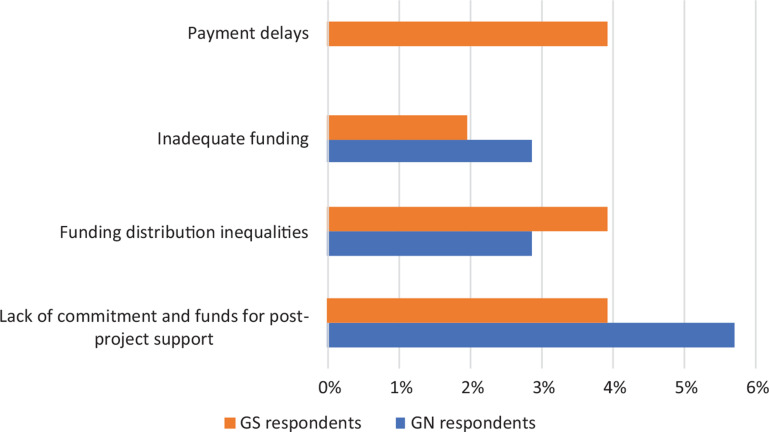
Financial challenges faced by survey respondents (% of respondents).

In the survey results, GN and GS respondents largely shared similar views about most of the financial difficulties associated with collaborative research projects: lack of funds for long-term project support, uneven distribution of funding between partners and inadequate funding (Fig. 3). A GN respondent explained how these factors can be particularly limiting for GS partners, highlighting that researchers from the GS often take on work in parallel to their research job, for international organisations like the International Energy Agency or for private sector firms. The respondent goes on to explain that, over time, this shifts their focus away from developing their skills and more towards earning enough to survive, which ultimately leads to limited contributions in their respective areas of research.

GS respondents also highlighted issues with payment delays. This could be due to the tendency for money to flow from GN institutions that usually secure the funding for their GS partners, adding extra administrative steps for the latter party. This is usually compounded by institutions in the GS not having the capacity to buffer delays in payments.

Another challenge flagged by researchers in the GS is a lack of resources. Research institutions in the GS tend to have limited capacity and support staff to help researchers engage in the proposal assembly process. For example, a workshop participant from Brazil remarked that his teaching duties take up most of his time and he has little time to dedicate to grant writing or engage in meaningful discussions to establish potential research partnerships. These capacity and contractual limitations have several consequences. The participant from Brazil, a senior academic in a local public university, for example, might not be eligible for salary costs as a co-investigator under current UKRI funding requirements. And resource constrained researchers are likely to focus their bidding efforts on grants with more lenient conditions and less paperwork as opposed to more competitive or reputable grants, which constrains their ability to do high-quality research and to participate in the international research community.

**Figure 4:**
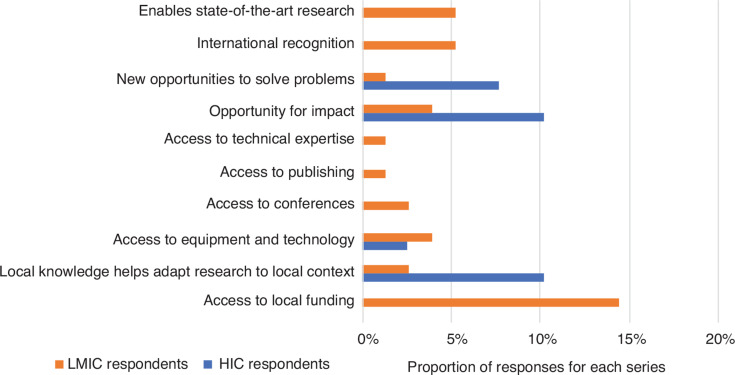
Resource challenges faced by survey respondents and opportunities provided by collaboration (% of respondents).

The survey results show that most respondents from the GS are motivated to engage in international research projects because of potential access to funding, while respondents from the GN mostly value the local knowledge of their partners in the GS that helps them adapt their research to the local context (Fig. 4). This point was well illustrated by a respondent from the GN, who stated that one major benefit from joint deliberation with researchers who have on-the-ground expertise is gaining a broader view of the research problem at hand.

Furthermore, the funds acquired from collaborative projects have a spill-over effect for GS researchers. One respondent from the GS explained that, through a recent collaboration, they were able to purchase equipment for their laboratory and acquire software licences.

### Research roles and power imbalances

**Figure 5:**
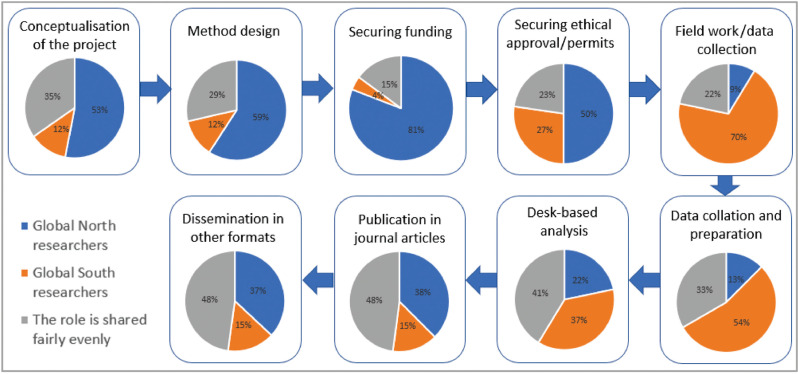
The roles of researchers from the GS and GN during different phases of project implementation.

The survey respondents were asked which parties – researchers from the GS, researchers from the GN or both – tend to dominate each stage of the research process. The findings (Fig. 5) suggest that GN partners dominate the conceptualisation, method design and grant proposal phases of collaborative research projects. Authorship of publications is shared between the two parties, but wider dissemination activities, such as presenting at conferences, are predominantly led by GN partners, possibly reflecting greater access to funding for travel and visa restrictions for GS researchers.

GS research partners, as anticipated, play a greater role in conducting fieldwork, data collection, data preparation and, to a lesser extent, desk-based analysis. This is very much in line with the literature on the subject – some studies have found that GS researchers normally conduct the core analysis for energy and development research in their geographies, but do not normally lead the journal publications that follow [8]. This ultimately yields more citations and academic recognition for the research partners in the GN in collaborative projects.

Workshop participants reported a similar sentiment with regards to research roles. This, among other things, can be attributed to funding stipulations for principal investigators to be based in Northern institutions. Some of the more senior researchers had experiences of this rigid hierarchical nature of funding, which, they believe, leads to unequal funding allocation. This was also characterised as a structural barrier because most research funding is provided by institutions in the GN. For example, while calls for proposals by UKRI requiring the principal investigator to be based in a UK research institution are understandable, it guarantees the involvement of the principal investigator in every stage of the project. The same is not true for collaborators from the GS who are often brought in after the grant has been awarded or during the data collection stage. By then, it is difficult for collaborators from the GS to contribute to the conceptual framework. It also means there are less opportunities for researchers from the GS to lead research projects and win grants, which are highly valuable skills.

**Figure 6:**
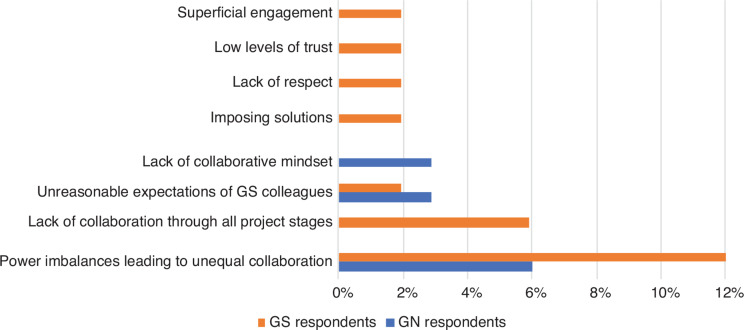
Perceived challenges linked to power imbalances between researchers in the GS and GN in collaborative research projects (% of respondents).

Challenges pertaining to power differentials were more frequently mentioned by GS respondents (Fig. 6). They noted the prevalence of power imbalances leading to unequal collaborations and the lack of collaboration through all project stages. They also reported lack of respect from their GN colleagues, frustrations with imposed solutions, low levels of trust between partners and superficial engagements. Some GN respondents also acknowledged similar issues. They felt that unreasonable expectations were sometimes asked of their GS colleagues and that there was sometimes a lack of collaborative mindset between parties.

### Context, research priorities and communication

**Figure 7:**
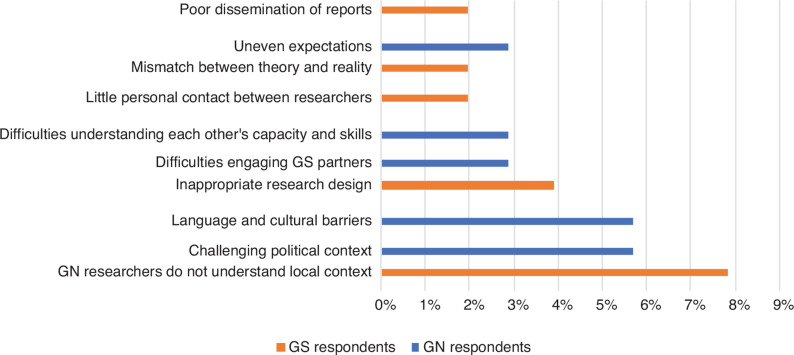
Contextual and communication challenges faced by survey respondents.

The next category of challenges concerned context and communication. GN and GS respondents identified different issues within this theme (Fig. 7).

GN researchers indicated that language differences, cultural barriers and challenging political contexts were the main barriers in collaborative projects. This is unsurprising as these projects involve GN researchers working in countries they are outsiders to. Some GN researchers also highlighted difficulties in engaging with and understanding GS research partners, which is indicative of tensions in collaborative research, potentially arising from imbalances and the cultural differences that neither side fully appreciate.

In the same vein, respondents from the GS identified their GN counterparts’ lack of understanding about the local context as the main challenge. This is problematic as it can lead to inappropriate research designs, which can have broader negative impacts on the project. This suggests that GN partners, who normally dominate the research design phase, may lack sufficient context-specific knowledge to tailor the research to the geography being studied. One GS respondent maintained that most of their collaborators from the GN start a research project with pre-determined rules of engagement without any knowledge of what is happening on the ground. On the contrary, one respondent from the GN highlighted this as a challenge that manifests due to communication barriers between researchers on either side of the divide.

Workshop participants emphasised that research agendas in the GN differ from those in the GS, and that funding often prioritises solving global challenges over local challenges, which leads to the import of global trends to the South while local priorities are overlooked. For example, one of the workshop participants observed that because the energy transition is viewed as a priority by academics in the GN it is automatically assumed to be a priority for GS researchers, a lot of whom view energy access as a more urgent matter. The fact that electricity access in some parts of the developing world is well below 50%, such as in most Sub-Saharan African countries [27], means that the idea of transitioning seems ludicrous to some local academics. Yet, when calls for proposals are centred around the energy transition, those same researchers have no option but to engage, and more likely, given the discussion thus far, in a secondary capacity.

Participants in the workshop stressed the importance of the environment within which researchers from the GS operate, such as the contextual knowledge held by GS research partners who live and work in the project’s country of study. Because of the necessity of local knowledge to navigate life in any country, GS researchers are often burdened with facilitating the travel and accommodation arrangements for visiting researchers from the GN (see also [20]). It is indeed ironic that local knowledge is deemed necessary for organising airport transfers but not when conceptualising research or designing funding calls. Another workshop participant from the GS mentioned that researchers in the GS often do invisible and unpaid labour, such as facilitating field visits. They are also often burdened with the menial, yet onerous, task of translating legal documents, contracts and surveys. Moreover, security issues in target regions introduce further complexity. Some participants who conduct research in ‘fragile’ states maintained that political instability makes international collaborations difficult as security risks sometimes compromise the data collection process and introduce doubts to funders about the likelihood of project completion.

### Collaboration and capacity building

When asked about the opportunities presented by engaging in collaborative projects, respondents from the GS did not recognise the increased possibility of solving problems or having an impact on the ground as much as respondents from the GN (Fig. 4). Instead, researchers from the GS highlighted, to a greater extent, the opportunities for international recognition they would not have had otherwise and engaging in state-of-the-art research. This viewpoint indicates that GS researchers see research practices in the GN as state-of-the-art. Given how some researchers from the GN view collaborations with GS researchers as necessary for gaining an understanding of the local context (see previous section) and, therefore, indispensable if the research is to be considered ‘state-of-the-art’, researchers from the GS clearly play an important role in any fruitful collaboration. But, perhaps because of deep-seated power imbalances or decades old research practices, GS researchers tend to underestimate their role.

Responses to the question on impact and recognition corroborate the hypothesis put forward by Ali et al. [8], that the methods applied in South–North research collaborations are normally determined by GN researchers. A respondent from the GS described that a general attribute of positive research collaborations is exposure to advanced research methods, tools and frameworks, and how these are incorporated into actionable policies. In contrast, a respondent from the GN claimed that what they find appealing in such collaborations is the chance to tackle real problems, progressing beyond superficial or theoretical work in the academic literature often conducted at an arm’s length. This difference in perspective could be due to the different environments within which researchers operate. GN researchers, because of resource availability, conducive institutions and stable environments, tend to operate at the boundaries of innovation, while researchers from the GS labour within contexts plagued by the ‘real problems’ GN researchers covet.

Finally, respondents from the GN and GS acknowledged that one major advantage of collaborative projects is the knowledge exchange that occurs in the process. Within this context, the themes identified by the respondents were more balanced between the two groups and included cultivating new networks, cultural exchanges, sharing knowledge and developing new friendships.

Capacity building – a process of individual and institutional development^2^ – was an important topic of discussion in the workshops. Some funding calls, such as UKRI’s GCRF, emphasise ‘capacity development’ ([26], p. 522) as a major component of funded projects, maintaining that it should be beneficial to all parties and designed to be long term. There was general agreement among workshop participants that capacity building should be a fundamental component of collaborative research projects, but that the concept should be unpacked to shed light on what capacity building entails for different parties. For example, who is being targeted by capacity building activities, what level of capacity development is proposed and how these activities are delivered are important considerations. A researcher from Sudan gave an example of a proposed collaborative research project between a government department in Sudan and a research organisation based in the US. The research proposed by the US-based organisation included capacity building as one of the main activities of the project but was limited to training enumerators on the latest data collection techniques. There was no mention of co-authorship of research outputs or engagement of the local research community in conceptualising the research questions. This experience was not unique and in the workshop was attributed to the limited funding and time allocated to capacity building in the design of collaborative research projects.

## Recommendations

In the final session of the workshop, participants were asked how collaborations between researchers in the GS and GN can be improved. The recommendations centred around three main themes: research agendas, research impact and capacity building. There were also institution-specific recommendations, mainly targeted at funding agencies, researchers and institutions and scientific journals.

On research agendas and impact, the workshop participants recommended that more effort should be made to link scientific research with local policy development and impact (as opposed to purely in pursuit of new evidence). They highlighted the urgent need to bridge the gap between global and local research agendas. Yet, this should be done while acknowledging the differences in contexts of the GS and GN, and how this may require different approaches or solutions. Within the GS, the differences between the numerous countries and regions should be recognised and blanket approaches should be avoided. For funding agencies, the workshop participants saw an urgent need to involve researchers from the GS in the early stages of developing funding calls and thought that local researchers should lead efforts to determine research agendas and priorities in their respective regions. Participants recommended that funding agencies should commit to research equity between GS and GN researchers in collaborative projects. This can be achieved by allowing principal investigators from GS institutions, offering pre-funding budgets to GS institutions to facilitate proposal development, and allocating equal amounts of funding between GS and GN institutions.

Similarly, capacity building efforts should recognise the structural and environmental limitations within which GS researchers operate. There is an ever-growing need for institutions in the GS to create a conducive environment for graduate students and junior researchers to develop. To ensure certain levels of capacity development, funding agencies could require the participation of local researchers in all stages of the project and co-authorship of publications and other outputs. Funders could both ensure and encourage long-term collaborations by requiring that research teams demonstrate already-existing relationships for large funding calls.

Researchers and institutions also have a responsibility to improve their research practices in collaborative projects. Researchers should put more effort into looking for, engaging with and citing literature authored by researchers in the GS, which may be published in less reputable journals and in a different language. In a similar vein, GN researchers should consider disseminating project outputs in languages other than English, and this should be reflected in extra funding for translation and dissemination. Moreover, before starting a collaborative research project, an open conversation should be held between the parties involved to set common and realistic rules of engagement to avoid imbalances in the research process. Processes should ensure that GN researchers involve their GS counterparts in all aspects of the project development process, from grant writing to conceptual development. Institutions have a responsibility of inculcating graduate students involved in collaborative research projects on equitable research processes. Similarly, institutions in the GN should assume responsibility for understanding the different pressures in GS institutions and make the necessary accommodations. Imbalances can be addressed through the contracting process, which should instil equity and balance between research partners. Lastly, institutions can make use of conference events by allocating more funding to facilitate travel for researchers from the GS to bring researchers together to build collaborations in-person.

Finally, workshop participants emphasised the need to include researchers in the GS in all stages of the process of disseminating research findings. This is an issue that has already been flagged in the social sciences; see, for example, Amarante et al. [29] and Chelwa [30], who show evidence of underrepresentation of developing country scholars and imbalances in authorship and editorial board membership in both Development Studies and Economics. There is an urgent need to include researchers from the GS in editorial boards of journals and in other stages of the peer-review process. This could help to encourage and support the dissemination and publication efforts of authors from the GS.

## Conclusions

The aim of this research was to identify the main challenges and opportunities of South–North collaborations in energy and development research, and to co-produce recommendations for key actors in the collaboration process.

This research brought forth key positive and negative aspects of South–North collaborations. The positive aspects, highlighted by both survey respondents and workshop participants, included obtaining a broader perspective on local issues and gaining access to resources and state-of-the-art research methodologies. The negative aspects centred around the persistent power imbalances in the research process, including agenda setting, unequal participation in different stages of the project, dominance of global challenges over local concerns, lack of communication between Southern and Northern researchers, lack of funding and capacity building opportunities for Southern researchers, and inequality in the conduct of publications.

Notably, the differences between challenges raised by participants reinforce the view that Northern perspectives dominate energy policy research, which does not necessarily improve or lead to reasonable policy guidelines. The application of methods and frameworks established by Northern institutions to propose policy to the GS, for instance, is usually an adaptation of existing knowledge and marginally benefits from local knowledge, risking inefficient policy recommendations.

On the one hand, the findings of this study agree with the dependency theorists’ claims that colonial history has produced a global political economy that retains the GS in the position of dependency [31]. The South’s subservient position produces, and is further exacerbated by, geopolitics of knowledge production. While dependency theorists would call for structural changes in global political economy to resolve this, the participants of this research suggested it is more realistic to tackle injustices within the resource and power constraints of those engaged in undertaking energy and development research. In effect, this research supplements calls for the geopolitics of knowledge production to be taken into account and for active measures that provide a more equitable position for Southern researchers [32].

Changing the political economy of knowledge production is a formidable challenge, as institutions in the GN hold the funds that support so many energy research projects. This means that the rules of engagement are often defined following Northern perspectives in keeping with Northern priorities. The actions recommended would provide small steps towards rectifying the power and resource imbalances in South–North research collaborations. The end goal of this effort, and others like it, is to ensure that energy policies implemented in developing countries are relevant and just. And we hope that, by highlighting existing discrepancies in the research process, we will help improve the quality of research that ultimately influences decisions on energy policy.

## Data Availability

The datasets generated during and/or analysed during the current study are available from the corresponding author on reasonable request.

## Notes

1 Parachute and helicopter research refer to an extractive approach to research, where researchers from higher-income countries conduct research, extract data and samples from lower-income countries with little involvement of local researchers and without appropriately acknowledging the importance of local expertise.

2 The definition of capacity building in this context is based on research capacity building, a concept widely adopted in medical and health research, defined as ‘a process of individual and institutional development which leads to higher levels of skills and greater ability to perform useful research’ ([28]: p. 1321).

## Appendix

The questions used in the survey to collect data on respondent experiences in South–North collaboration projects are listed below.

**Table:**

Question	Response
Q1: What is your main country of residence? (Select one)	List of countries
Q2: What is the main country, or countries, of focus for your research? (Select up to 4)	List of countries [Multiple selection]
Q3: At what kind of institution are you based? (Select one)	Select one of the following:
	Government
	Independent researcher/consultant
	International organisation
	Non-governmental organisation
	Public utility
	University
Q4: How is your research mainly funded?	
Q5: Where is the funding body based?	
Q6: Approximately how many research projects have you been involved in that involve a collaboration between researchers, or research institutions in the Global South (GS) and Global North (GN)?	
Q7: In what capacity have you generally been involved in these collaborations?	
Q8: Generally, has the principal investigator been based in the GS or GN? (Select one)	Select one of the following:
	GS
	GN
Q9: Broadly speaking, how has your experience been with the South–North research collaborations that you have been involved in?	
Q10: From your experience, what have been positive aspects of these collaborations? (900 characters max)	
Q11: From your experience, what have been negative aspects of these collaborations? (900 characters max)	
Q12: In general, who has dominated each of the following stages of the collaborative research projects you have been involved in:	For each stage, select one of the following:
	GN researchers
	GS researchers
	The role is shared fairly evenly
• [Conceptualisation of the project]	
• [Method design]	
• [Securing funding]	
• [Securing ethical approval and/or research permits]	
• [Field work/Data collection]	
• [Data collation and preparation]	
• [Desk-based analysis]	
• [Publication of results in journal articles]	
• [Publication/dissemination of results in other formats]	
Q13: If you have been involved in several collaborative projects, do you think they are generally more or less equitable? (Select one)	Select one of the following:
	I am seeing no change
	I am unable to say
	They are becoming more equitable
	They are becoming less equitable
Q14: How do you think collaborations between researchers in the GS and GN could be improved? (900 characters max)	
If you are interested in participating in the workshop, please provide your email address here.	
I consent to the use of information collected through this survey.	Select one of the following:
	Yes
	No
